# Nodal failure patterns and utility of elective nodal irradiation in submandibular gland carcinoma treated with postoperative radiotherapy - a multicenter experience

**DOI:** 10.1186/s13014-018-1130-y

**Published:** 2018-09-21

**Authors:** Cheng-En Hsieh, Li-Yu Lee, Yung-Chih Chou, Kang-Hsing Fan, Ngan-Ming Tsang, Joseph Tung-Chieh Chang, Hung-Ming Wang, Shu-Hang Ng, Chun-Ta Liao, Tzu-Chen Yen, Ku-Hao Fang, Chien-Yu Lin

**Affiliations:** 1 Radiation Oncology, Chang Gung Memorial Hospital at Linkou and Chang Gung University, No. 5, Fuxing St., Guishan Dist, Taoyuan City, 33305 Taiwan, Republic of China; 2 Pathology, Chang Gung Memorial Hospital at Linkou and Chang Gung University, Taoyuan City, Taiwan, Republic of China; 3 Medical Oncology, Chang Gung Memorial Hospital at Linkou and Chang Gung University, Taoyuan City, Taiwan, Republic of China; 4 Diagnostic Radiology, Chang Gung Memorial Hospital at Linkou and Chang Gung University, Taoyuan City, Taiwan, Republic of China; 5 Otorhinolaryngology, Head and Neck Surgery, Chang Gung Memorial Hospital at Linkou and Chang Gung University, Taoyuan City, Taiwan, Republic of China; 6 Nuclear Medicine and Molecular Imaging Center, Chang Gung Memorial Hospital at Linkou and Chang Gung University, Taoyuan City, Taiwan, Republic of China; 7 Head and Neck Oncology Group, Chang Gung Memorial Hospital at Linkou and Chang Gung University, Taoyuan City, Taiwan, Republic of China; 8 Graduate Institute of Clinical Medical Science, Chang Gung University, Taoyuan City, Taiwan, Republic of China; 9 School of Traditional Chinese Medicine, Chang Gung University, Taoyuan City, Taiwan, Republic of China; 10 Radiation Oncology, Xiamen Chang Gung Hospital, Xiamen City, Fujian Province China; 11 Departments of Experimental Radiation Oncology, Division of Radiation Oncology, The University of Texas MD Anderson Cancer Center, Houston, Texas USA; 12 The University of Texas MD Anderson Cancer Center-UT Health Graduate School of Biomedical Sciences, Houston, Texas USA; 13 Particle Physics and Beam Delivery Core Laboratory, Institute for Radiological Research, Chang Gung Memorial Hospital at Linkou and Chang Gung University, Taoyuan City, Taiwan, Republic of China

**Keywords:** Submandibular gland cancer, Postoperative radiotherapy, Elective nodal irradiation, Nodal failure pattern

## Abstract

**Background:**

The patterns of nodal relapse in submandibular gland carcinoma (SMGC) patients treated with postoperative radiotherapy (PORT) remain unclear. This study aims to investigate the nodal failure patterns and the utility of elective nodal irradiation (ENI) in SMGC patients undergoing PORT.

**Methods:**

We retrospectively enrolled 65 SMGC patients who underwent PORT between 2000 and 2014. The nodal failure sites in relation to irradiation fields and pathological parameters were analyzed. ENI regions were categorized into three bilateral echelons (first, levels I–II; second, level III; and third, levels IV–V). Extended ENI was defined as coverage of at least the immediately adjacent uninvolved echelons bilaterally; otherwise, limited ENI was administered.

**Results:**

Thirty patients (46%) were stage III–IV, and 18 (28%) were pN+. Neck irradiation included limited (72%) and extended ENI (28%). With a median follow-up of 79 months, 11 patients (17%) developed nodal failures (ipsilateral, *N* = 6; contralateral, *N* = 7), 7 (64%) of whom relapsed in the adjacent uninvolved echelons. We identified pN+ (*P* = 0.030), extranodal extension (ENE, *P* = 0.002), pT3–4 (*P* = 0.021), and lymphovascular invasion (LVI, *P* = 0.004) as significant predictors of contralateral neck recurrence. Extended ENI significantly improved regional control (RC) in patients with pN+ (*P* = 0.003), ENE (*P* = 0.022), pT3–4 (*P* = 0.044), and LVI (*P* = 0.014), and improved disease-free survival (DFS) in patients with pN+ (*P* = 0.034). For patients with ≥2 coincident adverse factors, extended ENI significantly increased RC (*P* < 0.001), distant metastasis-free survival (*P* = 0.019), and DFS (*P* = 0.007); conversely, no nodal recurrence was observed in patients without these adverse factors, even when only the involved echelon was irradiated.

**Conclusions:**

Nodal failure is not uncommon in SMGC patients treated with PORT if pN+, ENE, pT3–4, and LVI are present. Extended ENI should be considered, particularly in patients with multiple pathological adverse factors.

**Electronic supplementary material:**

The online version of this article (10.1186/s13014-018-1130-y) contains supplementary material, which is available to authorized users.

## Background

Submandibular gland carcinomas (SMGCs) are rare malignancies, accounting for less than 10% of salivary gland neoplasms and 1% of head and neck cancers [1–3]. *En bloc* radical resection is the mainstay of treatment [4], and postoperative radiotherapy (PORT) has been utilized in patients with adverse pathological factors including advanced stage, high-grade tumors, positive surgical margins, bone invasion, and perineural invasion (PNI) [5–9]. The behavior of SMGC and the optimal PORT treatment remain unclear. Data on combined-modality treatments usually encompass parotid gland cancer patients, and the nodal failure patterns and clinical utility of elective nodal irradiation (ENI) for SMGC remain vague [6, 10–13]. The currently recommended nodal irradiation fields are based on the treatment experience of different salivary gland malignancies (most of them arising from the parotid gland).

The incidence of neck metastases in parotid gland carcinoma is reportedly 12–25% [6, 8, 11, 14, 15], with no risk to the contralateral neck [11, 16]. However, the submandibular gland has more extensive lymphatic drainage; hence, SMGC is more amenable to nodal metastasis [6, 10, 17]. According to a detailed study by the Dutch Head and Neck Oncology Group, pathologically positive nodes were detected in 42% of SMGC patients, and the nodal metastasis rate approached 60% for high-grade and advanced T-stage tumors [6]. Even when a unique histology entity (adenoid cystic carcinoma, ACC) is considered, the rates of nodal metastases from submandibular gland malignancies are higher than those observed for parotid gland tumors (22.5% vs 14.5%, respectively) [18]. Furthermore, increased risks of contralateral neck metastasis (12.4-fold) and occult nodal metastasis have been reported in oral cavity squamous cell carcinoma that directly invades into the floor of the mouth [19–21]. As the submandibular glands are proximal to the floor of the mouth and midline of the neck, it is unclear whether contralateral neck treatment should be omitted as it is for parotid gland cancer patients. Therefore, we conducted this retrospective multicenter study to review the long-term outcomes of SMGC patients treated with radical resection and PORT. Additionally, we investigated the use and treatment outcomes of ENI, and identified patients who would most benefit from extended ENI.

## Methods

### Patients and clinical workup

A total number of 74 patients with SMGC treated with radical surgery and PORT at Linkou, Kaohsiung, Keelung, and Chiayi Chang Gung Memorial Hospitals between January 2000 and December 2014 were identified. Patients with distant metastasis at diagnosis (*N* = 5), history of prior irradiation (*N* = 3), and lymphoma histology (*N* = 1) were excluded; 65 patients were ultimately enrolled. The staging workups and follow-up schedules were described previously [22]. The cancer staging was revised according to the seventh edition (2010) of the American Joint Committee on Cancer staging criteria, and the tumor histology was defined according to 2005 World Health Organization classification.

### Treatment

**Fig. 1:**
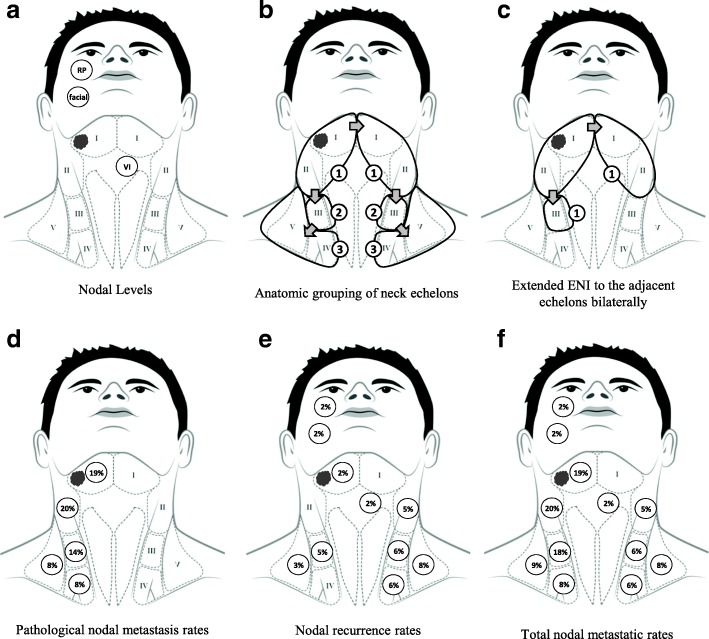
**a** Depiction of each of Level I–V, retropharyngeal (RP) and facial nodes. **b** The neck irradiation regions were categorized into 3 consecutive bilateral echelons according to lymphatic drainage: the first echelon for levels I–II, the second for level III, and the third for levels IV–V. **c** Taking a submandibular gland carcinoma (SMGC) patient with multiple risk factors as an example, if the tumor invades level I, extended elective nodal irradiation (ENI) of the adjacent bilateral echelons involves the ipsilateral levels I–III and contralateral levels I–II. **d** Pathological nodal metastatic rates in 24 patients who received ipsilateral neck dissection. **e** Nodal recurrent rates and (**F**) total nodal metastatic rates in 65 SMGC patients treated with surgery and postoperative radiotherapy

All subjects were treated with radical submandibulectomy, and neck dissection was performed for those with clinical nodal involvement or locally advanced tumors. PORT was administered using megavoltage photon irradiation, 1.8–2 Gy per fraction, five times per week using either three-dimensional conformal radiotherapy, intensity-modulated radiation therapy, or volumetric modulated arc therapy delivery systems. The prophylactic dose of ENI was 46–50 Gy with a 60–66 Gy boost to high-risk regions. ENI was generally administered to patients presenting with adverse pathological features. The neck irradiation regions were categorized into 3 consecutive bilateral echelons according to the lymphatic drainage anatomy of the submandibular gland: the first echelon was for levels I–II, the second for level III, and the third for levels IV–V (Fig. 1a, b). Extended ENI encompassed the adjacent uninvolved echelons bilaterally according to the pathological tumor extension (Fig. 1c). Otherwise, limited ENI, defined as the irradiation of the involved echelon alone or ipsilateral ENI for adjacent uninvolved echelons without prophylactic contralateral neck treatment, was administered. Concurrent chemotherapy was generally applied for patients with adverse pathological features [23]; intravenous cisplatin was the most commonly used agent (typically at 100 mg/m^2^ once every 3 weeks or 40 mg/m^2^ once per week) [24, 25].

### Statistical analysis

Intergroup differences in continuous variables were tested using independent Student’s *t*-tests. Categorical data were compared using the Pearson’s chi-squared or the Fisher’s exact test, as appropriate. Local control (LC), regional control (RC), distant metastasis-free survival (DMFS), disease-free survival (DFS), and overall survival (OS) were calculated from the date of surgery to the date of detection of the relevant events, respectively. Survival curves were plotted using the Kaplan-Meier method and compared using the log-rank test. Multivariate analysis was performed using Cox proportional hazards regression models with a stepwise forward conditional manner. Variables were retained in the model if their significance levels were < 0.05. Results are expressed as hazard ratios with their 95% confidence intervals. All data analyses were performed using the SPSS 20.0 statistical software (IBM Corporation, Armonk, New York, USA).

## Results

### Patient characteristics

Patient characteristics are demonstrated in Table 1. The median age was 53 years, and the most common histological subtypes were ACC, carcinoma ex pleomorphic adenoma, and mucoepidermoid carcinoma.

**Table 1:** Patient characteristics

Characteristic	*N*	%
Sex		
Female/Male	35/30	54/46
Age (years)		
Median (range)	53 (24–79)	
Performance score		
ECOG 0–1	65	100
T stage		
pT1	15	23
pT2	24	37
pT3	20	31
pT4a	6	9
Tumor size (cm)		
Median (range)	2.9 (0.5–8.0)	
N stage		
pN0	6	9
pN1	3	5
pN2b	15	23
cN0/pNx^a^	41	63
Disease stage		
I	13	20
II	22	34
III	11	17
IVa	19	29
Staging modality		
CT	49	75
MRI	6	9
^18^F-FDG-PET	23	35
Surgical margin		
< 1 mm/≥1 mm	46/19	71/29
Histology		
Adenoid cystic carcinoma	29	45
Carcinoma ex pleomorphic adenoma	11	17
Mucoepidermoid carcinoma	8	12
Lymphoepithelial carcinoma	6	9
Squamous cell carcinoma	4	6
Salivary duct carcinoma	4	6
Adenocarcinoma	2	3
Myoepithelial carcinoma	1	2
Histology grading		
Low to intermediate/High	6/59	9/91
Pathological features		
Perineural invasion	35	54
Extranodal extension	12	19
Bone invasion	2	3
Skin invasion	1	2
Lymphovascular invasion	18	28
Neck dissection		
None	41	63
Ipsilateral elective	9	14
Ipsilateral therapeutic	15	23
Contralateral	0	0
< 18 nodes	22	34
≥18 nodes	17	26
Nodal irradiation		
Extended ENI	18	28
Limited ENI	47	72
Concurrent chemotherapy	24	37
Radiotherapy technique		
3D-CRT	19	29
IMRT	31	48
VMAT	15	23
Radiotherapy dose (Gy)		
Median (range)	66 (32–72)	

Abbreviations: *3D-CRT* three-dimensional conformal radiotherapy, *CT* computed tomography, *ECOG* Eastern Cooperative Oncology Group, *ENI* elective nodal irradiation, ^*18*^*F-FDG-PET* 18F-fluorodeoxyglucose positron emission tomography, *IMRT* intensity-modulated radiation therapy, *MRI* magnetic resonance imaging, *VMAT* volumetric modulated arc therapy

^a^,no elective neck dissection in cN0 patients

Clinically positive nodes were observed in 15 patients (23%); 24 patients (37%) received ipsilateral neck dissection while none received contralateral neck surgery. Consequently, pathological nodal metastasis was detected in 18 patients (28%). The distribution of pathological positive nodes is illustrated in Fig. 1d.

### Adjuvant treatments

The median cumulative radiation dose was 66 Gy, and only one patient received a dose less than 50 Gy due to poor health. The median time from surgery to PORT initiation was 34 (range, 11–71) days; the median time was 35 (range, 11–71) days and 29 (range, 14–49) days among those with limited ENI and extended ENI, respectively. Concurrent chemoradiotherapy was administered to 24 patients (limited ENI, *N* = 16; extended ENI, *N* = 8), the most commonly used regimen was intravenous cisplatin-based (*N* = 23). Single-agent cisplatin was administered to 18 patients, whereas concurrent oral uracil-tegafur and cisplatin were administered to the remaining 5 patients. The median cumulative cisplatin dose was 200 mg/m^2^. Sixteen of the 23 patients (70%) completed their planned chemotherapy course; one patient received concurrent cetuximab treatment. Neither neoadjuvant nor adjuvant chemotherapy was administered.

### Neck irradiation fields

Extended ENI was performed in 18 patients (28%), covering the next adjacent uninvolved echelons bilaterally (*N* = 8) or ≥ 2 additional echelons (*N* = 10). Otherwise, limited ENI was performed in the remaining 47 patients; the irradiated neck regions consisted of involved echelons alone in 30 patients (46%) and of ipsilateral adjacent uninvolved echelons in 17 (26%). Notably, 12 of these patients received unintentional irradiation doses to the contralateral levels I (*N* = 7) or I/II (*N* = 5) that encountered the radiation beam paths.

### Treatment outcomes and nodal failure patterns

The median follow-up time for the survivors was 79 (range, 19–183) months. By the end of the study, 21 patients (32%) had died owing to cancer recurrence in 16 and intercurrent diseases in five (coronary artery disease, *N* = 2; pneumonia, *N* = 2; and cerebral hemorrhage, *N* = 1). Three patients developed secondary malignancies (gum squamous cell carcinoma, ovarian adenocarcinoma, and cholangiocarcinoma, respectively); the patient with secondary cholangiocarcinoma died of uncontrolled tumor bleeding resulting from SMGC locoregional recurrence (patient #11), and the two other patients were alive with controlled secondary malignancies. The five- and 10-year OS rates were 72 and 63% for the entire cohort, respectively.

Cancer recurrence was recorded in 21 patients (32%). The five- and 10-year DFS rates were 64 and 64%, respectively. The predominant form of treatment failure was distant metastasis (20 patients), and the five- and 10-year DMFS rates were both 66%. Three patients had local recurrence; among them, perineural tumor recurrence was recorded in two ACC patients who were treated with surgical bed irradiation alone (hypoglossal nerve, *N* = 1, #11; facial and lingual nerves, *N* = 1, #12; Table 2, Additional file 1: Figure S1). When we investigated the highest margin of the irradiation fields, we identified that coverage up to the transverse process of the first cervical vertebrae (for level II neck coverage only), mastoid tip, and skull base were performed in 10 (35%), 1 (3%), and 18 (62%) patients while 2 (20%), 0 (0%), and 0 (0%) had outfield recurrence (*P* = 0.180). The five- and 10-year LC rates 96 and 92%, respectively.

**Table 2:**
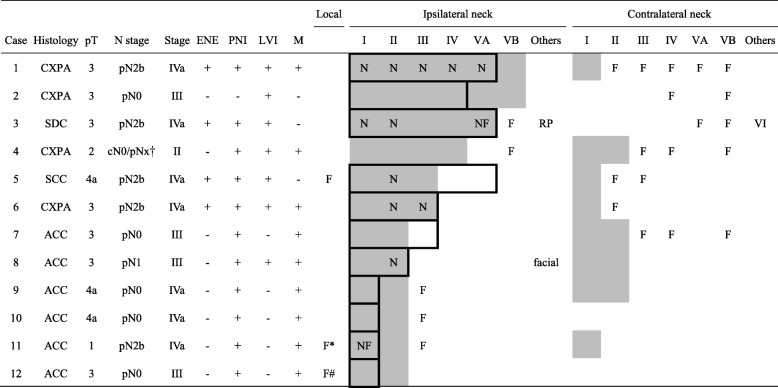
Neck irradiation fields and failure patterns in 12 submandibular gland carcinoma patients who developed locoregional recurrence after postoperative radiotherapy

Gray screentones: areas of neck irradiation. Frames: areas of nodal dissection

Abbreviations: *ACC* adenoid cystic carcinoma, *ENE* extranodal extension, *CXPA* carcinoma ex pleomorphic adenoma, *F* site of failure, *LVI* lymphovascular invasion, *M* surgical margins, *N* pathologically involved nodal region, *PNI* perineurial invasion, *pT* pathological T stage, *RP* retropharyngeal nodal failure, *SCC* squamous cell carcinoma, *SDC* salivary duct carcinoma

†, no elective neck dissection in cN0 patients; Perineural tumor recurrence at *hypoglossal nerve (Additional file 1: Figure S1B) and #facial and lingual nerves (Additional file 1: Figure S1A).

Eleven patients (17%) experienced neck recurrence, with five- and 10-year RC rates of 80% and 80%, respectively. The median time to nodal recurrence was 25 (range, 6–57) months in 10 patients with limited ENI and 52 months in 1 patients with extended ENI. Ipsilateral and contralateral neck failures were documented in six and seven patients, respectively; the distributions of recurrent nodes and total nodal metastatic rates are shown in Fig. 1e-f. Seven patients (64%) developed nodal recurrence in the adjacent uninvolved echelons. The detailed neck nodal failure sites and corresponding radiation fields are shown in Table 2. Ten of the 11 patients who experienced nodal relapse also developed uncontrolled distant metastases.

### Univariate and multivariate analyses of adverse pathological factors

On univariate analysis (Table 3), the presence of pN+, ENE, pT3–4, LVI, and PNI was significantly correlated with inferior RC, LC, DMFS, and DFS. A significantly worse five-year LC rate was observed in patients with pN+. The presence of pN+, ENE, pT3–4, and LVI was each a significant predictor of poorer OS.

**Table 3:** Univariate and multivariate analyses of adverse pathological factors

*Univariate analysis*		RC (%)		LC (%)		DMFS (%)		DFS (%)		OS (%)	
Variable	*N*	5-year	*P*	5-year	*P*	5-year	*P*	5-year	*P*	5-year	*P*
N stage											
pN+	18	49.6	0.002	75.0	0.015	31.1	< 0.001	24.5	< 0.001	37.5	< 0.001
pN0 & cN0/pNx^a^	47	87.7		100		77.5		77.8		85.2	
Extranodal extension											
Yes	12	55.4	0.004	83.3	0.149	21.2	< 0.001	21.8	< 0.001	30.0	< 0.001
No	53	84.2		97.3		74.2		72.0		81.0	
T stage											
pT3–4	26	57.9	< 0.001	93.8	0.104	33.2	< 0.001	35.1	< 0.001	45.9	< 0.001
pT1–2	39	93.1		96.7		85.5		82.2		88.9	
Lymphovascular invasion											
Yes	15	51.0	< 0.001	90.0	0.569	29.6	< 0.001	30.5	< 0.001	44.4	< 0.001
No	50	89.4		96.9		78.6		75.0		82.7	
Perineural invasion											
Yes	35	66.2	0.007	92.0	0.084	48.9	0.002	45.7	0.001	66.3	0.092
No	30	95.7		100		87.9		87.9		78.0	
Margin < 1 mm											
Yes	47	79.1	0.910	96.6	0.794	68.7	0.418	66.0	0.449	75.7	0.475
No	18	80.9		91.7		57.0		57.5		61.1	
High-grade histology											
Yes	59	77.1	0.204	94.9	0.466	63.0	0.324	61.3	0.287	68.4	0.079
No	6	100		100		85.7		85.7		100	
*Multivariate analysis*											
Variable		HR	95% CI		*P*	Variable			HR	95% CI	*P*
Regional control						Disease-free survival					
T stage (pT3–4 vs pT1–2)		8.129	1.661–39.779		0.010	N stage (pN+ vs pN0 & cN0/pNx^a^)			4.026	1.464–11.075	0.007
Lymphovascular invasion (Yes vs No)		4.130	1.165–14.643		0.028	Perineural invasion (Yes vs No)			5.377	1.512–19.121	0.009
Local control		–	–		NS	T stage (pT3–4 vs pT1–2)			3.762	1.262–11.213	0.017
Distant metastasis-free survival						Overall survival					
T stage (pT3–4 vs pT1–2)		5.171	1.715–15.591		0.004	N stage (pN+ vs pN0 & cN0/pNx^a^)			4.363	1.710–11.127	0.002
Perineural invasion (Yes vs No)		4.337	1.234–15.236		0.022	T stage (pT3–4 vs pT1–2)			4.841	1.671–14.020	0.004
Lymphovascular invasion (Yes vs No)		2.719	1.065–6.942		0.037						

Abbreviations***:*** *CI* confidence interval, *DFS* disease free survival, *DMFS* distant metastasis free survival, *HR* Hazard ratio, *LC* local control, *NS* not statistically significant, *OS* overall survival, *RC* regional control

^a^,no elective neck dissection in cN0 patients

Multivariate analyses revealed that pT3–4 and LVI significantly correlated with poorer RC. No statistically significant prognosticator for LC was identified (Table 3). Notably, pT3–4, LVI, and PNI were identified as significant independent predictors of DMFS, whereas pN+, PNI, and pT3–4 were significantly correlated with worse DFS; pN+ and pT3–4 were significant predictors of inferior OS.

### Predictors of nodal recurrence

Neither univariate nor multivariate analyses revealed significant predictors of ipsilateral nodal recurrence; only a trend toward worse ipsilateral nodal failure was observed in patients with pN+ or pT3–4 (Table 4). However, pN+, ENE, pT3–4, and LVI were significant predictors of contralateral neck recurrence on univariate analysis; whereas ENE and LVI were significant independent prognosticators of the same on multivariate analysis.

**Table 4:** Univariate and multivariate analyses of clinicopathological risk factors for neck failure

*Variables*	Ipsilateral neck failure						Contralateral neck failure					
	Univariate analysis			Multivariate analysis			Univariate analysis			Multivariate analysis		
	HR	95% CI	*P*	HR	95% CI	*P*	HR	95% CI	*P*	HR	95% CI	*P*
N stage (pN+ vs pN0 & cN0/pNx^a^)	4.491	0.888–22.717	0.069	–	–	NS	5.305	1.176–23.925	0.030	–	–	NS
Extranodal extension (Yes vs No)	1.607	0.185–13.984	0.667	–	–	NS	11.479	2.519–52.305	0.002	6.018	1.203–30.104	0.029
T stage (pT3–4 vs pT1–2)	5.102	0.903–28.819	0.065	–	–	NS	12.293	1.472–102.668	0.021	–	–	NS
Lymphovascular invasion (Yes vs No)	3.738	0.751–18.597	0.107	–	–	NS	22.007	2.643–183.267	0.004	14.920	1.694–131.385	0.015
Perineural invasion (Yes vs No)	63.645	0.089–45,483	0.215	–	–	NS	5.274	0.635–43.827	0.124	**–**	**–**	NS
Margin (< 1 mm vs ≥ 1 mm)	1.860	0.217–15.951	0.571	–	–	NS	0.441	0.099–1.974	0.285	**–**	**–**	NS
High-grade histology (Yes vs No)	24.834	0.001–872,589	0.548	–	–	NS	24.431	0.001–526,351.9	0.530	**–**	**–**	NS
Ipsi. elective neck dissection (Yes vs No)	0.039	0.000–876.767	0.526	–	–	NS	–	–	–	–	**–**	–
Ipsi. elective nodal irradiation (Yes vs No)	0.402	0.073–2.203	0.294	–	–	NS	–	–	–	–	**–**	–
Cont. elective nodal irradiation (Yes vs No)	–	–	–	–	–	–	0.778	0.151–4.013	0.764	**–**	**–**	NS

Abbreviations***:*** *CI* confidence interval, *Cont*. contralateral, *HR* hazard ratio, *Ipsi*. ipsilateral, *NS* not statistically significant

^a^, no elective neck dissection in cN0 patients

### Extended elective nodal irradiation

**Fig. 2:**
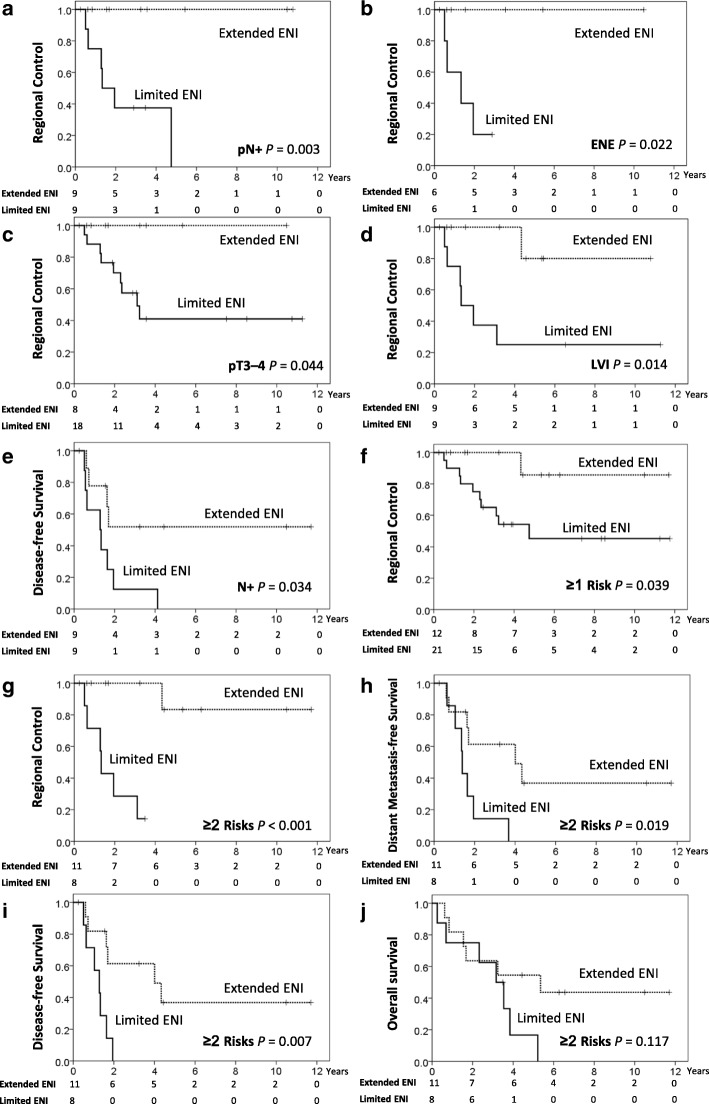
**a**–**d** Regional control curves in patients with pN+, extranodal extension (ENE), pT3–4, and lymphovascular invasion (LVI); and (**e**) disease-free survival curves in pN(+) patients; treated with extended vs limited elective nodal irradiation (ENI). **f** Regional control curves in patients with ≥1 adverse factors, and (**g**–**j**) regional control, distant metastasis-free survival, disease-free survival, and overall survival curves in patients with ≥2 adverse factors treated with extended or limited ENI. (Adverse factors: pN+, ENE, pT3–4, and LVI)

To identify specific populations of SMGC patients who might benefit from extended ENI, we performed subgroup analyses to examine each pathological parameter. As demonstrated in Table 5 and Fig. 2a-d, extended ENI significantly improved RC rates for patients with pN+ (*P* = 0.003), ENE (*P* = 0.022), pT3–4 (*P* = 0.044), and LVI (*P* = 0.014). For patients with pN+, we observed a significant improvement in five-year DFS in those treated with extended ENI (52% vs 0%, *P* = 0.034, Fig. 2e). Although higher five-year DMFS rates were observed for pN+ patients treated with extended ENI (52% vs 13%, *P* = 0.166), as well as higher five- and 10-year OS rates (44 and 44% vs 28 and 0%, *P* = 0.333), the differences were not significant.

**Table 5:** Survival outcomes of submandibular gland carcinoma patients bearing adverse pathological risk factors, treatment with or without postoperative bilateral elective nodal irradiation

Variable		RC (%)			DMFS (%)			DFS (%)			OS (%)		
	*N*	5-y	10-y	*P*	5-y	10-y	*P*	5-y	10-y	*P*	5-y	10-y	*P*
Entire cohort													
Extended ENI	18	90.0	90.0	0.207	59.3	59.3	0.700	59.3	59.3	0.871	70.3	61.6	0.636
Limited ENI	47	76.6	76.6		67.6	67.6		65.4	65.4		72.3	63.6	
pN+													
Extended ENI	9	100	100	0.003	51.9	51.9	0.166	51.9	51.9	0.034	44.4	44.4	0.333
Limited ENI	9	0	–		12.5	–		0	–		27.8	0	
Extranodal extension													
Extended ENI	6	100	100	0.022	44.4	44.4	0.328	44.4	44.4	0.161	50.0	50.0	0.319
Limited ENI	6	20.0	–		0	–		0	–		0	–	
pT3–4													
Extended ENI	8	100	100	0.044	40.0	40.0	0.694	40.0	40.0	0.579	37.5	18.8	0.288
Limited ENI	18	41.0	41.0		32.7	32.7		34.3	34.3		49.1	32.8	
Lymphovascular invasion													
Extended ENI	9	80.0	80.0	0.014	32.4	32.4	0.472	32.4	32.4	0.351	55.6	44.4	0.392
Limited ENI	9	25.0	25.0		25.0	25.0		25.0	25.0		33.3	22.2	
Perineural invasion													
Extended ENI	9	80.0	–	0.353	25.9	–	0.195	25.9	–	0.300	55.6	41.7	0.270
Limited ENI	26	66.3	66.3		57.0	57.0		52.6	52.6		69.4	52.9	
Margin < 1 mm													
Extended ENI	12	83.3	83.3	0.512	61.1	61.1	0.905	61.1	61.1	0.763	71.4	53.6	0.374
Limited ENI	35	78.0	78.0		70.3	70.3		66.8	66.8		77.1	65.4	
High grade histology													
Extended ENI	41	88.9	88.9	0.179	63.3	63.3	0.899	63.3	63.3	0.650	68.4	58.6	0.721
Limited ENI	17	73.1	73.1		63.0	63.0		60.4	60.4		68.6	58.8	
0 risk factor^a^													
Extended ENI	6	100	100	–	100	100	0.533	100	100	0.533	100	100	0.449
Limited ENI	26	100	100		91.1	91.1		91.1	91.1		87.9	87.9	
≥1 risk factor^a^													
Extended ENI	12	85.7	85.7	0.039	43.2	43.2	0.558	43.2	43.2	0.349	58.3	48.6	0.752
Limited ENI	21	45.1	45.1		38.2	38.2		31.5	31.5		52.4	32.7	
≥2 risk factors^a^													
Extended ENI	11	83.3	83.3	< 0.001	36.8	36.8	0.019	36.8	36.8	0.007	54.5	43.6	0.117
Limited ENI	8	14.3	–		0	0		0	0		16.7	0	

Abbreviations: *DFS* disease free survival, *DMFS* distant metastasis free survival, *ENI* elective nodal irradiation, *OS* overall survival, *RC* regional control

^a^***Risk factors:*** pN+, extranodal extension, pT3–4, and lymphovascular invasion

To identify the subgroups of patients who might benefit the most from extended ENI, we stratified patients according to pN+, ENE, LVI, and pT3–4 (the patient characteristics between the treatment groups are listed in Additional file 2: Table S1). In patients with ≥1 adverse pathological factor(s), extended ENI to ≥1 adjacent echelons significantly increased five- and 10-year RC rates (86 and 86% vs 45 and 45%, respectively, *P* = 0.039; Table 5 and Fig. 2f). However, the DMFS, DFS, and OS rates did not significantly differ between each group. For patients with ≥2 coincident adverse pathological factors, extended ENI to the adjacent echelons bilaterally increased five-year RC (83% vs 14%, *P* < 0.001, Fig. 2g), DMFS (37% vs 0%, *P* = 0.019, Fig. 2h), and DFS (37% vs 0%, *P* = 0.007, Fig. 2i) rates significantly. However, the five- and 10-year OS rates were not significantly higher in patients treated with extended ENI (55 and 44% vs 16.7 and 0%, respectively; *P* = 0.117, Fig. 2j). On the other hand, the five- and 10-year RC rates were 100% and 100% in patients who did not receive extended ENI (*N* = 26) but had none of the aforementioned adverse pathological factors; neither elective nodal dissection nor irradiation of the adjacent echelons was performed in 14 of these patients. Furthermore, no significant differences were observed in terms of RC, DMFS, DFS, and OS rates in these low-risk patients whether treated with extended ENI or not.

## Discussion

Despite their generally indolent clinical course, nodal and distant recurrences remain a major clinical concern in SMGC patients who carry adverse pathological factors, even following combined-modality treatment [1, 2, 6, 8, 26]. The recommended ENI fields of PORT for SMGC derive from the indications outlined for parotid cancer, where ipsilateral level I–V irradiation is adopted for patients with advance T-stage, pN(+), high-grade histology, PNI, and recurrent disease. Ipsilateral upper neck ENI is recommended for cases with early high-risk tumors [6, 10–13].

However, the submandibular gland has a rich lymphocapillary network. A large surgical series revealed that submandibular malignancies had a significantly higher risk of occult nodal metastasis (21%) than parotid gland cancers (9%) [10]. In our previous study of salivary gland cancer patients treated with PORT, those with SMGC exhibited a significantly lower five-year RC rate (73.9%) compared to patients with tumors originating from other salivary glands (parotid, 91.2%; sublingual, 100%; and minor salivary, 100%) [23]. These data suggest that the nodal spreading behavior of SMGCs differs from that of parotid gland tumors, and more aggressive treatment may be warranted for select high-risk SMGC patients.

In the present study, the overall incidence of neck metastasis was as high as 35% (pN+, *N* = 18 [27%]; neck recurrence in patients with pN0 or cN0/pNx [i.e., no elective neck dissection performed for clinically negative necks], *N* = 5, [8%]; Fig. 1d–f) which is consistent with other series [1, 17, 27–29]. Occult metastasis was documented in 9% of our patients (pN+ in cN0 patients, *N* = 1; neck recurrence in pN0 or cN0/pNx, *N* = 5). Our data indicated that contralateral nodal metastasis was not uncommon in SMGC patients (Fig. 1d), particularly in the presence of pN+, ENE, pT3–4, and LVI (Table 4), suggesting that ipsilateral ENI of the neck may be inadequate for these high-risk subgroups. However, our data did not identify predictors for ipsilateral neck relapse, possibly owing to the small number of events and the fact that ipsilateral neck prophylactic treatment was frequently performed for patients with adverse pathological factors. Importantly, we found that 64% of nodal recurrences were located in the adjacent uninvolved echelons, suggesting that ENI of the adjoining echelons might be warranted in selected patients. Notably, 10 of the 11 patients who experienced nodal relapse (91%) also developed distant metastases; this strong correlation implies that reducing nodal recurrence might in turn decrease the risk of distant failure.

Positive surgical margins are a poor prognostic factor and have been reported in 36–46% of SMGC patients [17, 28]. Compared to other surgical series, we observed a higher incidence of surgical margins < 1 mm (71%). This might be attributed to more conservative resections as well as the clinical aggressiveness of SMGC. Intriguingly, our data demonstrated that resection margins < 1 mm were not significantly associated with poor outcomes in the setting of adjuvant radiotherapy; this was comparable to our previous findings with parotid cancer [30]. This may indicate the high efficacy of adjuvant radiotherapy in eradicating microscopic tumors, especially as only two patients experienced nodal relapses within the initial irradiation fields.

We hypothesized that the anatomic proximity of SMGCs to the floor of the mouth and midline of the neck increases the risk of contralateral neck and occult metastasis, and that ipsilateral or limited ENI is inadequate in such cases. Therefore, we categorized the ENI fields into three consecutive bilateral echelons accordingly to anatomical lymphatic drainage. We found no statistically significant differences in disease control and survival rates between patients treated with or without extended ENI. However, in subgroup analyses, extended ENI significantly improved RC rates in patients exhibiting adverse pathological factors. In pN+ patients in particular, a significantly higher DFS rate was observed in those treated with extended ENI, suggesting that a reduced nodal failure rate might translate into a DFS benefit.

To identify SMGC patients who may benefit most from extended ENI, we stratified our patients according to the aforementioned adverse factors. For patients with ≥1 risk factor(s), bilateral ENI extension to the adjacent echelons significantly improved the five-year RC rate; however, there were no significant differences in DMFS, DFS, and OS rates between the groups. Importantly, for patients with ≥2 coincident risk factors, extended ENI significantly increased the five-year RC, DMFS, and DFS rates. The five- and 10-year OS rates were also (non-significantly) higher in patients treated with extended ENI, suggesting that patients bearing multiple coincident adverse factors may derive the most benefit from extended ENI. Conversely, for patients without any of the aforementioned risk factors, irradiation of the involved echelon alone appears to be sufficient for decreasing treatment-related morbidities.

Concordant with the published literature [17, 31], our multivariate analysis identified PNI as an independent predictor for worse DMFS and DFS, but not for inferior RC. Additionally, no significant RC improvement was observed in patients with perineural invasion treated with extended ENI, suggesting that the presence of PNI alone may not warrant the use of extended ENI. Nonetheless, the current study documented that 19 (66%) out of 29 ACC patients presented with PNI, and two (11%) of the 19 developed outfield perineural tumor recurrence. Since a higher propensity of perineural invasion/spread in ACC has been documented [32, 33], prophylactic irradiation to the nerve tract should be strongly recommended particularly in the presence of PNI and locally-advanced disease. However, the distance from the tumor at which radiation treatment should be administered cannot be determined by this limited data. Coverage of the nerve tract of the lingual nerve, hypoglossal nerve, and facial nerve (marginal mandibular branch) to the skull base might be adequate in preventing morbidity as a result of brain irradiation. Additionally, it is warranted to extend outwards to cover the angle of the jaw and the plane between the plasma for the marginal mandibular branch.

We observed a high nodal recurrent rate (17%) in SMGC patients treated with PORT. Additionally, failures at contralateral neck nodes and uninvolved adjacent echelons were frequently observed, particularly in patients with pN+, ENE, pT3–4, and LVI. To our knowledge, we are the first to demonstrate the indications and clinical utility of extended ENI for the bilateral treatment of adjacent echelons of SMGC. However, there are some limitations inherent to this retrospective study. While ours was the largest long-term series of SMGC patients treated with PORT, the relatively small number of subjects may have biased our results. The median follow-up of 79 months was relatively short for accurately evaluating the survival outcomes for SMGC. Additionally, we cannot fully account for all potential biases due to the non-randomized retrospective nature of the study. The subjective treatment decisions and the diverse irradiation fields used may also be regarded as limitations. However, because of the low prevalence and indolent nature of salivary gland malignancies, conducting a decade-long prospective randomized trial would be difficult. The only ongoing randomized trial, RTOG 1008, is designed to investigate the efficacy of postoperative cisplatin-based chemoradiation in patients with salivary gland carcinomas; the target enrollment size is 120 patients. Importantly, this trial does not specifically assess SMGC patients; the neck irradiation field for SMGC is based on empirical experience. Hence, we believe that our multicenter experience is valuable and provides a rationale for the design of future prospective trials.

## Conclusion

Our long-term study showed that nodal recurrence on the contralateral side of the neck and adjacent uninvolved echelons was not uncommon in SMGC patients treated with PORT, and that this should be considered during radiotherapy planning. Extended ENI appears to improve RC and DFS rates in patients with certain (or multiple) adverse pathological factors. Conversely, limited ENI to the involved echelons alone appears to be adequate for low-risk patients without risk factors.

## Additional files

Additional file 1: **Figure S1.** Perineural tumor recurrence at (A) lingual (solid arrow), inferior alveolar (arrow head) and facial (dashed arrow) nerves (#12) and (B) hypoglossal nerve (#11). (PDF 63 kb)

Additional file 2: **Table S1.** Patient characteristics in selected patients with risk factors. (DOCX 334 kb)
