# Supplementary figures and images for: Nodal failure patterns and utility of elective nodal irradiation in submandibular gland carcinoma treated with postoperative radiotherapy - a multicenter experience
Source: Radiat Oncol. 2018 Sep 21;13:184. doi: 10.1186/s13014-018-1130-y (PMC6151022; doi:10.1186/s13014-018-1130-y)

A

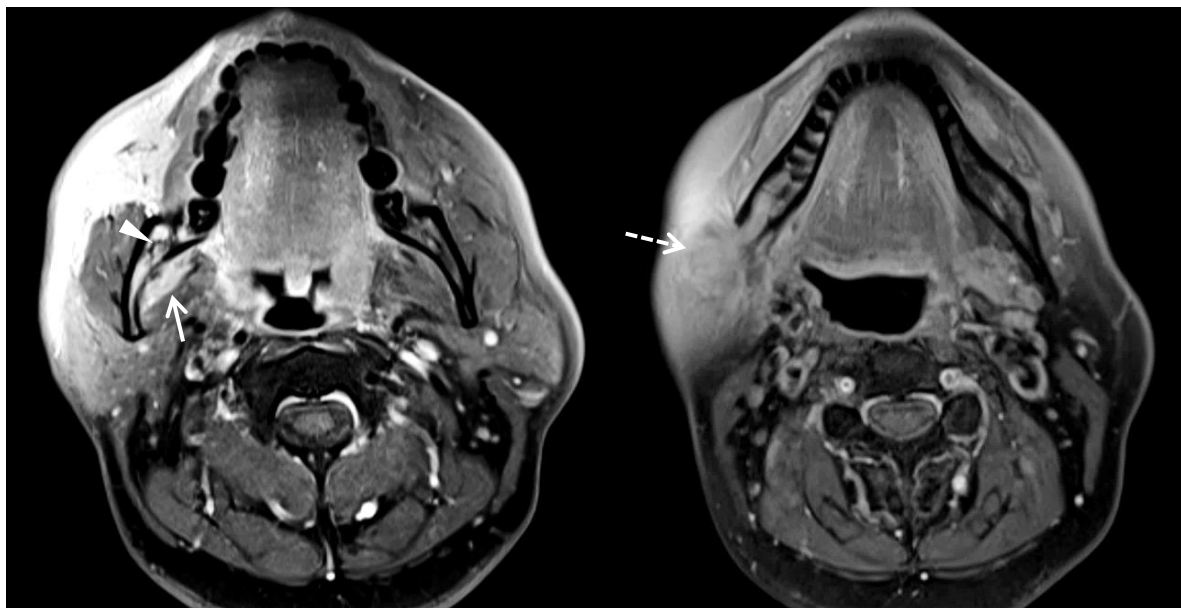

B

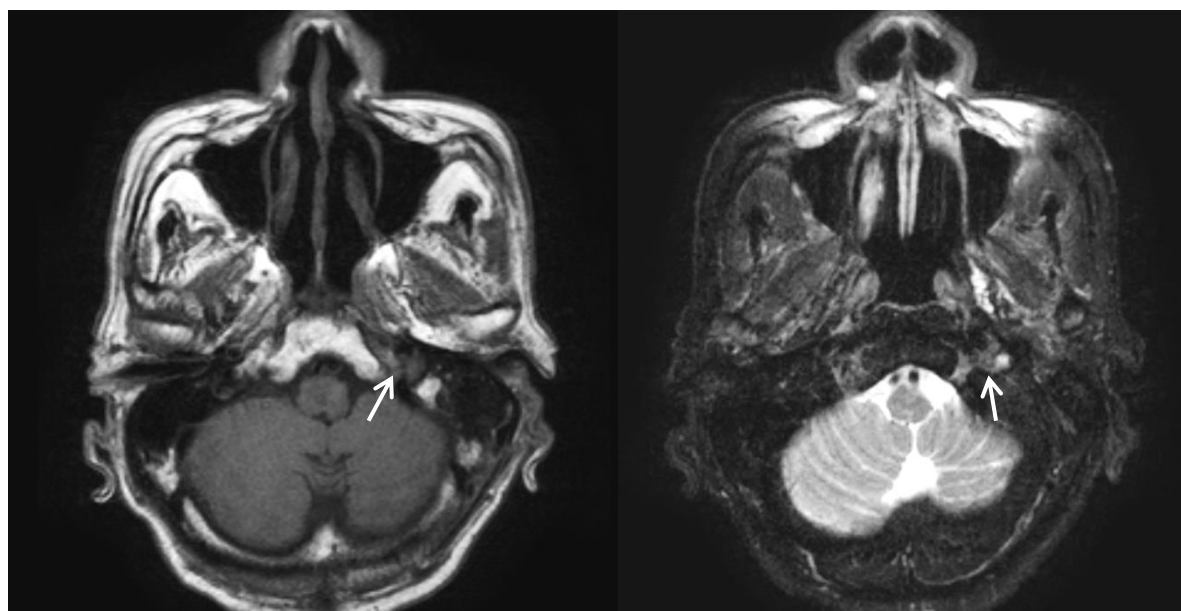

Supplement: Supplementary file 1 — Figure S1. Perineural tumor recurrence at (A) lingual (solid arrow), inferior alveolar (arrow head) and facial (dashed arrow) nerves (#12) and (B) hypoglossal nerve (#11). (PDF 63 kb) [file 13014_2018_1130_MOESM1_ESM.docx]
