# Supplementary material for: Nodal failure patterns and utility of elective nodal irradiation in submandibular gland carcinoma treated with postoperative radiotherapy - a multicenter experience
Source: Radiat Oncol. 2018 Sep 21;13:184. doi: 10.1186/s13014-018-1130-y (PMC6151022; doi:10.1186/s13014-018-1130-y)
Supplement: Supplementary file 2 — Table S1. Patient characteristics in selected patients with risk factors. (DOCX 334 kb) [file 13014_2018_1130_MOESM2_ESM.pdf]

**Additional file 2**

**Table S1.** Patient characteristics in selected patients with risk factors

|  |  | **≥1 risk factor*** | | |  | **≥2 risk factors*** | | |
| --- | --- | --- | --- | --- | --- | --- | --- | --- |
|  | **ENI volume** | **Limited** | **Extended** |  |  | **Limited** | **Extended** |  |
| **Variable** |  | ***N* (%)** | ***N* (%)** | ***P*** |  | ***N* (%)** | ***N* (%)** | ***P*** |
| **Sex** | Female | 11 (52) | 5 (42) | 0.721 |  | 6 (75) | 4 (36) | 0.170 |
|  | Male | 10 (48) | 7 (58) |  |  | 2 (25) | 7 (64) |  |
| **Age** **(years)** | ≥ 65 | 5 (24) | 1 (8) | 0.379 |  | 3 (38) | 1 (9) | 0.262 |
|  | < 65 | 16 (76) | 11 (92) |  |  | 5 (62) | 10 (91) |  |
| **T stage** | pT1-2 | 3 (14) | 4 (33) | 0.377 |  | 0 (0) | 3 (27) | 0.228 |
|  | pT3-4 | 18 (86) | 8 (67) |  |  | 8 (100) | 8 (73) |  |
| **N stage** | pN0 | 8 (38) | 0 (0) | **0.025** |  | 1 (13) | 0 (0) | 0.659 |
|  | cN0/pNx* | 5 (24) | 3 (25) |  |  | 1 (13) | 2 (18) |  |
|  | pN1 | 0 (0) | 2 (17) |  |  | 0 (0) | 2 (18) |  |
|  | pN2b | 8 (38) | 7 (58) |  |  | 6 (74) | 7 (64) |  |
| **Disease stage** | I-II | 2 (10) | 2 (17) | 0.610 |  | 0 (0) | 1 (9) | 1.000 |
|  | III-IV | 19 (90) | 10 (83) |  |  | 8 (100) | 10 (91) |  |
| **Staging modality** | CT | 14 (67) | 11 (92) | 0.206 |  | 5 (63) | 10 (91) | 0.262 |
|  | MRI | 3 (14) | 0 (0) | 0.284 |  | 1 (13) | 0 (0) | 0.421 |
|  | ^18^F-FDG-PET | 7 (33) | 5 (42) | 0.716 |  | 3 (38) | 4 (36) | 1.000 |
| **Surgical margin** | <1 mm | 15 (71) | 7 (58) | 0.471 |  | 4 (50) | 7 (64) | 0.658 |
| **Histology** | Adenoid cystic carcinoma | 7 (33) | 3 (25) | 0.682 |  | 1 (13) | 2 (18) | 0.703 |
|  | Carcinoma ex pleomorphic adenoma | 4 (19) | 3 (25) |  |  | 3 (38) | 3 (27) |  |
|  | Mucoepidermoid carcinoma | 4 (19) | 1 (8) |  |  | 1 (13) | 1 (9) |  |
|  | Lymphoepithelial carcinoma | 2 (10) | 2 (17) |  |  | 0 (0) | 2 (18) |  |
|  | Squamous cell carcinoma | 2 (10) | 1 (8) |  |  | 2 (25) | 1 (9) |  |
|  | Salivary duct carcinoma | 1 (5) | 0 (0) |  |  | 1 (13) | 0 (0) |  |
|  | Adenocarcinoma | 0 (0) | 2 (17) |  |  | 0 (0) | 2 (18) |  |
| **Histology grading** | High grade | 19 (90) | 12 (100) | 0.523 |  | 8 (100) | 11 (100) | 1.000 |
| **Pathological features** | Perineural invasion | 13 (62) | 9 (75) | 0.703 |  | 6 (75) | 8 (7) | 1.000 |
|  | Extranodal extension | 6 (29) | 6 (50) | 0.274 |  | 6 (75) | 6 (55) | 0.633 |
|  | Bone invasion | 1 (5) | 1 (8) | 1.000 |  | 1 (13) | 1 (9) | 1.000 |
|  | Skin invasion | 0 (0) | 1 (8) | 1.000 |  | 0 (0) | 1 (9) | 1.000 |
|  | Lymphovascular invasion | 9 (43) | 9 (75) | 0.145 |  | 7 (88) | 8 (73) | 0.603 |
| **Neck dissection** | None | 5 (24) | 3 (25) | 0.420 |  | 1 (13) | 2 (18) | 0.603 |
|  | <18 nodes | 8 (38) | 2 (17) |  |  | 0 (0) | 2 (18) |  |
|  | ≥18 nodes | 8 (38) | 7 (58) |  |  | 7 (87) | 7 (64) |  |
| **Concurrent chemotherapy** | Yes | 8 (38) | 6 (50) | 0.716 |  | 3 (38) | 6 (55) | 0.650 |
|  | No | 13 (62) | 6 (50) |  |  | 5 (62) | 5 (45) |  |
| **Radiotherapy technique** | 3D-CRT | 6 (29) | 4 (33) | 1.000 |  | 1 (13) | 4 (36) | 0.555 |
|  | IMRT | 12 (57) | 6 (50) |  |  | 5 (62) | 6 (55) |  |
|  | VMAT | 3 (14) | 2 (17) |  |  | 2 (25) | 1 (9) |  |
| **Radiotherapy dose (Gy)** | Mean ± SD | 62 ± 8 | 66 ± 2 | 0.093 |  | 60 ± 13 | 66 ± 2 | 0.216 |
| **Interval from surgery to PORT (d)** | Mean ± SD | 35 ± 12 | 28 ± 9 | 0.088 |  | 36 ± 13 | 28 ± 10 | 0.132 |
|  | Median (range) | 34 (15-62) | 28 (14-49) |  |  | 34 (19-62) | 28 (14-49) |  |

****Risk factors:*** pN+, extranodal extension, pT3–4, and lymphovascular invasion.

***Abbreviations:*** 3D-CRT, three-dimensional conformal radiotherapy; CT, computed tomography; ECOG, Eastern Cooperative Oncology Group; ENI, elective nodal irradiation; ^18^F-FDG-PET, 18F-fluorodeoxyglucose positron emission tomography; IMRT, intensity-modulated radiation therapy; MRI, magnetic resonance imaging; VMAT, volumetric modulated arc therapy; PORT, postoperative radiotherapy; *, no elective neck dissection in cN0 patients.
